# Case Report: Constraint led strength and conditioning for Paralympic throwers with skeletal dysplasia

**DOI:** 10.3389/fspor.2025.1677491

**Published:** 2025-10-27

**Authors:** Lawrence W. Judge, Exal Garcia-Carrillo

**Affiliations:** ^1^ Marieb College of Health & Human Services, Florida Gulf Coast University, Fort Meyers, FL, United States; ^2^ School of Kinesiology, Ball State University, Muncie, IN, United States; ^3^ Department of Physical Activity Sciences, Universidad de Los Lagos, Osorno, Chile; ^4^ Department of Physical Activity Sciences, Faculty of Education Sciences, Universidad Católica del Maule, Talca, Chile

**Keywords:** athletic performance, resistance training, mental health, psychological resilience, disabled persons, sports for persons with disabilities, adaptive sports, para-athletes

## Abstract

**Introduction:**

Paralympic athletes with skeletal dysplasia (SD) require specialized strength and conditioning approaches due to unique anthropometric characteristics. This study examines how a constraints-led model optimizes training in an elite shot-putter with short stature.

**Methods:**

A case study of a F41 shot-put Paralympic medalist (height: 147 cm; body-mass: 72 kg) through biomechanical analysis and training programming. The intervention combined: modified resistance training addressing joint instability, sport-specific plyometrics adapted for shorter limbs, and environmental adaptations for equipment accessibility. Performance metrics included throwing distance, strength-to-mass ratio, and kinematic measurements of shot-put release.

**Results:**

Implementation yielded a 13.88 m competition throw (Tokyo 2021 Paralympics), with maintained body fat of 12%–14%. Results were achieved without training-related injuries, highlighting the model's efficacy in balancing performance enhancement with joint preservation.

**Conclusions:**

A constraints-based approach to strength and conditioning, combined with dynamic periodization and a comprehensive support system, provides an adjustable framework for optimizing performance in Paralympic athletes with SD. This multifaceted approach ensures that both physical and psychological demands are met, enabling sustainable athletic development while accounting for the unique biomechanical and physiological characteristics of athletes with SD. Future research should focus upon refining the application of these principles in diverse adaptive sports to further enhance performance outcomes and reduce injury risks.

## 1 Introduction

Effective coaching, often regarded as an art, must be firmly grounded in a robust scientific foundation, especially when addressing the unique needs of Paralympic athletes (1). This population faces distinct physiological and psychological challenges, making a science-based approach crucial for optimizing performance and well-being (2). The constraints-led model of skill acquisition offers a comprehensive and adaptable framework for strength and conditioning programming, enabling the development of highly individualized and context-specific training interventions (3), which may prove particularly beneficial for athletes with skeletal dysplasia (SD). SD, a heritable bone disorder mainly characterized by short stature, is defined as height three or more standard deviations below the mean for age and sex (4). This condition includes over 400 distinct etiologies, such as metabolic, endocrine, congenital, and chromosomal abnormalities, each presenting distinct challenges in athletic training (5). The condition's prevalence, estimated at 1 in 5,000 births (6), underscores the necessity of early diagnosis and targeted intervention strategies. Among the various forms of SD, achondroplasia is the most common non-lethal variant, while others like achondrogenesis and thanatophoric dysplasia are typically fatal (7, 8).

Adaptive sports have been proven to offer both health and psychological benefits and has dramatic impact on a population such as para-athletes allowing them to overcome barriers and challenges (9). Studies have documented significant improvements in physical quality of life and moderate gains in mental quality of life following engagement in adaptive sports (10). In addition to these standardized metrics, participants report subjective enhancements in strength, balance, mood and self-efficacy following their participation in adapted programs (10), these interventions help overcome physical and social barriers by providing tailored equipment, community support, and psychologically safe environments where athletes are not defined by their condition. This synergy between psychosocial support and technological accommodation is foundational. Equipment modifications (e.g., tailored prostheses, sport-specific wheelchairs) are not merely functional, they signal inclusivity, bridging the gap between ability and opportunity (11). Likewise, training accommodations (e.g., adjusted facilities, rule modifications) are structured to help para-athletes reach their optimal potential by aligning practice with individualized requirements (12). Together, these adaptations prioritize safety while maximizing performance, creating a holistic framework for athletic development.

Hagan Landry, 26 years old at the time of the Tokyo Games, with a stature of 147 cm and mass of 72 kg, competed in the F41 short-stature sport class. His profile offers a compelling case study for the constraints-led model of Paralympic strength and conditioning. Landry captured the silver medal in men's shot-put with an American-record throw of 13.88 m at the Tokyo Paralympic Games in 2021, (estimated release angle: 38.5°; speed: 11.7 m/s; height: 1.55 m). This release height represents a ≈30%–34% reduction compared to able-bodied throwers (typically 2.20–2.35 m) (13), yet was compensated by release speed, demonstrating effective adaptation to SD constraints. Throughout the 2020 macro-cycle he maintained a 12%–14% body-fat range (≈8.8–10.3 kg of fat mass), mirroring the 12.9% benchmark documented in longitudinal studies of elite able-bodied shot-putters and comfortably below the 15%–18% mean reported for national-level male throwers (14). This favorable composition augmented his strength-to-mass ratio, a key predictor of shot-put performance in power athletes (15). Landry's preparation, directed by an interdisciplinary staff, systematically harmonized individual constraints (SD–related limb proportions and joint kinetics), environmental constraints (modified lifting stations and variable-height throwing platforms), and task constraints (F41-specific implement weight and release-angle adjustments) in accordance with contemporary constraints-led coaching literature. This paper synthesizes the case-study season blueprint into a reproducible framework for coaches of Paralympic throwers, integrating diagnostic assessments, dynamically undulating periodization, and empirically derived body-composition targets. This project was reviewed and approved by the Ball State University Institutional Review Board under protocol #812324-1. All procedures involving human participants conformed to the ethical standards of the institution and to the principles of the Declaration of Helsinki. Written informed consent was obtained from all participants prior to data collection, and participant data were anonymized before analysis to protect confidentiality.

## 2 Understanding constraints in athletic performance

Constraints are critical determinants that influence motor learning and athletic performance, particularly in dynamic and complex environments. These constraints can be categorized into three primary domains: individual, environmental, and task-related factors (12). For athletes with SD, each domain requires specialized consideration due to their unique physiological and biomechanical challenges. Individual constraints, such as joint instability, disproportionate limb, and muscular imbalances, necessitates the modification of traditional training programs to accommodate altered biomechanics and reduce injury risk. Environmental factors, including accessibility to specialized equipment and adaptive training environments, are vital in facilitating effective strength and conditioning programs. Task-related constraints, such as modifications in sport-specific rules and equipment to suit athletes' physical capabilities, must be integrated into their training regimens (16). Understanding the interplay between these constraints allows for the development of individualized, context-specific training interventions that optimize performance while addressing the specific physical and psychological needs of athletes with SD. Table 1 outlines the Constraints-Based Approach to Strength and Conditioning Programming and Periodization for Paralympic Athletes with SD, integrating individual, task-specific, environmental, and psychological constraints into program design. These considerations ensure that training programs are tailored to the unique characteristics of athletes, enhancing performance while minimizing injury risk.

**Table 1:** A constraints-based approach to training paralympic athletes with skeletal dysplasia (SD).

Constraint type	Description	Application in strength & conditioning for athletes with SD	Examples in training
Individual Constraints	Refers to the athlete's unique physical, psychological, and functional characteristics.	Training must consider limitations in joint mobility, muscle strength imbalances, and skeletal structure differences associated with SD.	Perform modified exercises to accommodate reduced range of motion (e.g., partial squats or supported overhead presses).
Task Constraints	Refers to the specific demands of the sport or activity being trained for.	Shot-put performance requires development of explosive power, rotational strength, and proper hip extension.	Use lighter loads with explosive exercises like medicine ball throws, emphasizing hip rotation and extension movements.
Environmental Constraints	The external environment, including training facilities, equipment, and competition settings.	Facilities may need to be adapted to accommodate accessibility challenges. Athletes with SD may require specialized equipment for safety.	Ensure accessible gym layouts and adjustable equipment (e.g., adjustable benches or lifting platforms for shorter limbs).
Periodization Constraints	Refers to how the training program is organized over time to maximize performance while avoiding injury.	Athletes with SD may need longer recovery periods and careful monitoring of load to avoid joint stress and injury.	Implement a flexible periodization plan with lighter loading phases and built-in recovery days to prevent overuse injuries.
Psychological Constraints	Mental aspects, such as motivation, self-confidence, and coping with performance anxiety.	Psychological factors must be addressed to help the athlete maintain motivation and confidence despite physical challenges.	Incorporate motivational strategies and mental resilience training (e.g., visualization techniques for improving performance).

## 3 Mitigating risks

Working with Para-athletes has an increased level of injury risks (17), and accounting for this allows coaching to not only be efficient but also aids in maximizing performance. Injuries to athletes with disabilities or co-morbidities such as the ones present in the Dwarfism community may have a repercussion on functionality (18). Using additional equipment or tools to best suit the athlete can create a beneficial practice environment, while also maximizing efficiency. Using technology such as wearable sensors such as EMG sensors, to identify the motor control that an athlete possesses (19). Heart rate sensors could also be a resource on monitoring the training load exerted while utilizing a labor-intensive practice to avoid overexertion and fatigue (19). Monitoring risks and taking extra precautions only fosters a healthy environment for the athlete to physically exceed and mentally prosper.

## 4 Individualized assessment and programming

To effectively implement a constraints-based approach, each Paralympic athlete should undergo a comprehensive assessment that accounts for their unique physical, psychological, and biomechanical characteristics (11). This multidimensional evaluation includes not only traditional strength and conditioning metrics but also individualized performance diagnostics aligned with the athlete's classification and impairment profile. Tailoring programming to these data ensures that exercises and training modalities remain both effective and safe, promoting sustainable long-term athletic development (16). Hagan Landry's performance team was led by his coach, who orchestrated integration among sport scientists, strength & conditioning coaches, biomechanists, medical staff, and other specialists, ensuring each domain contributed cohesively to his preparation.

For athletes with short stature (SD), specific assessments are critical to evaluate factors such as joint stiffness, bone fragility, muscle imbalances, and altered biomechanics. These variables have direct implications for force production and technical execution. Identifying such constraints allows for targeted interventions that improve performance capacity while minimizing injury risk (20). Psychological assessments further complement this process, helping to identify key factors such as motivation, confidence, and resilience—attributes that directly influence training adherence and competitive readiness (21). Rayes emphasizes that participation in adaptive sport enhances not only physical performance but also psychological well-being and quality of life (22).

In this context, the Overhead Back (OHB) shot-put test using a 4 kg implement was employed as a periodic fitness checkpoint throughout the 24-week mesocycle leading to the Tokyo 2021 Paralympic Games. The OHB test served as an integrated field-based measure of neuromuscular power, coordination, and general readiness. Progressive increases in throw distance, from 12.00 m at baseline to 14.10 m five days before competition, reflected cumulative strength and power adaptations while also providing valuable feedback on fatigue and potential overtraining. When performance plateaus or regressions were observed, training load adjustments were made to restore optimal recovery. Thus, the OHB functioned as both a diagnostic and preventive tool within the athlete's individualized monitoring framework, complementing formal strength assessments and subjective wellness indices.

Translating these assessments into actionable programming required a structured, mesocycle-based training plan designed to enhance performance while mitigating the unique risks associated with SD. By integrating individualized data, including biomechanical assessments, psychological profiling, and OHB performance trends, coaches could precisely calibrate training volume, intensity, and recovery to optimize power, strength, and mobility while minimizing injury risk (23). This evidence-based, adaptive framework ensures that programming not only maximizes competitive readiness but also supports long-term athlete health, resilience, and performance sustainability (12). As shown in Table 2, the athlete's Overhead Back (OHB) shot-put results over a 24-week build-up illustrate systematic gains in throw distance, with trends monitored to flag any signs of overtraining or fatigue.

**Table 2:** Overhead back shot-put results (lead-up to Tokyo 2021, F41).

Test Interval	Date	Throw distance (m)	*Δ* from previous (m)	Cumulative *Δ* (m)	Change (%)	Notes/conditions
Baseline (pre)	2021-03-25	12.00	—	0.00	—	Initial test
4 weeks	2021-04-22	12.31	+0.31	+0.31	+2.58%	
8 weeks	2021-05-20	12.22	−0.09	+0.22	+1.83%	
12 weeks	2021-06-17	13.40	+1.18	+1.40	+11.67%	
16 weeks	2021-07-15	13.73	+0.33	+1.73	+14.42%	
20 weeks	2021-08-12	14.02	+0.29	+2.02	+16.83%	
24 weeks (pre-final)	2021-08-25	14.10	+0.08	+2.10	+17.50%	Final test (5 days before)

Final test five days before competition, competition on 30 August 2021.

Competition held 30 August 2021; final test conducted 25 August 2021 (5 days prior).

## 5 Three-day training program for paralympic shot-put athlete with SD

This mesocycle-based program is designed to optimize key performance variables in Paralympic shot-put, focusing on power, absolute strength, hypertrophy, hip extension, and rotation. The program incorporates plyometrics, such as drop jumps, which are known to enhance power and throwing performance (23). For a shot-putter with SD, exercises have been carefully selected to improve explosive power while minimizing joint strain, with a focus on enhancing rotational strength and hip extension to compensate for potential mobility limitations while generating maximum force during the shot-put throw.

This program (Table 3), when executed three times a week, is structured to allow sufficient recovery and adaptation, which is crucial for optimizing strength, muscle hypertrophy, and hip extension/rotation. It is specifically tailored to meet the unique needs and capabilities of the athlete with SD.

**Table 3:** Training program for paralympic shot-Put athlete with SD.

Day	Focus	Warm-up	Main exercises	Core work
Day 1 (Monday)	Upper Body Hypertrophy	Hang clean complex (mid-thigh high pulls, mid-thigh cleans, hip cleans): 5 × 3	• Dumbbell power jerks: 4 × 5 • Bench press (65% 1 RM): 4 × 12 • Band pull-aparts: 3 × 12 • Bench dips: 5 × 12 • Seated rows: 5 × 12 • Medicine ball drops 3 × 5	Russian twist: 3 × 5
Day 2 (Wednesday)	Absolute Strength	5 min cycling + knee cleans: 3 × 5	• Below-knee snatch pulls: 7 × 2 • Hang power cleans (thigh level): 6 × 2 • Front squats: 8 × 3	Bicycle crunches: 3 × 10
Day 3 (Friday)	Hip Extension and Rotation	Push-press with light weights (emphasizing bar speed)	• Rocket jumps (18-inch box): 10 × 1 • Back hyperextensions with plate: 5 × 15 • Backward Russian twists on hyper bench (with power bar or similar): 5 × 20	–

Exercise notation follows sets × repetitions format.

1 RM, one-repetition maximum; all percentage-based loads were calculated from 1 RM values.

## 6 Environmental adaptations

Para-athletes often encounter barriers beyond the social standpoint but their training environment as well. Coaches must ensure that training environments are fully accessible, inclusive, and tailored to meet the specific needs of athletes with SD. This includes adaptive equipment, accessible facilities, and curating an inclusive environment that values diversity in athletic abilities (24). Athletes with SD, environmental adaptations are essential to facilitate effective and safe training, particularly when accounting for their unique anthropometric constraints.

**Figure 1:**
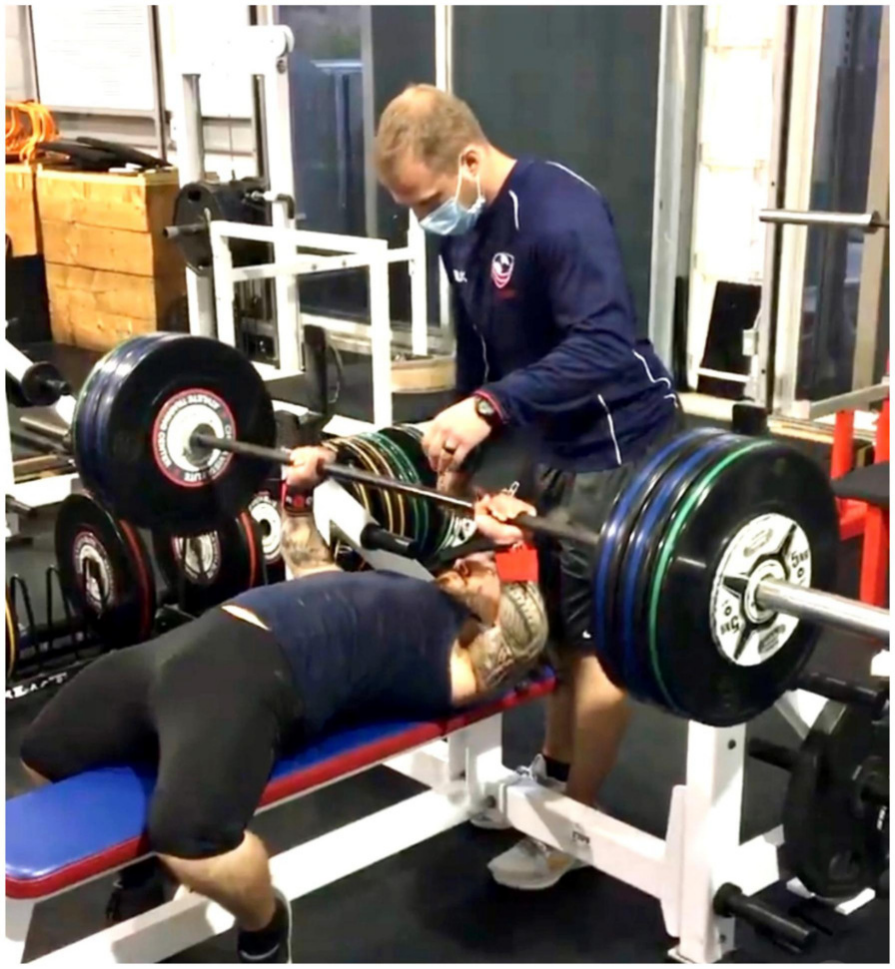
Bench-press adaptation for an F41 paralympic thrower. Hagan Landry (classification F41; standing height  ≈ 1.38 m) performs a flat bench press with each foot placed on a 15 cm plyometric box. Elevating the feet enables full-surface plantar contact, recreating the leg-drive mechanics used by average-stature athletes while keeping hip and knee angles near 90°. The boxes also provide a stable anchor to counter posterior translation of the torso at heavy loads, thereby preserving scapular positioning and minimizing shear stress on the lumbar spine.

**Figure 2:**
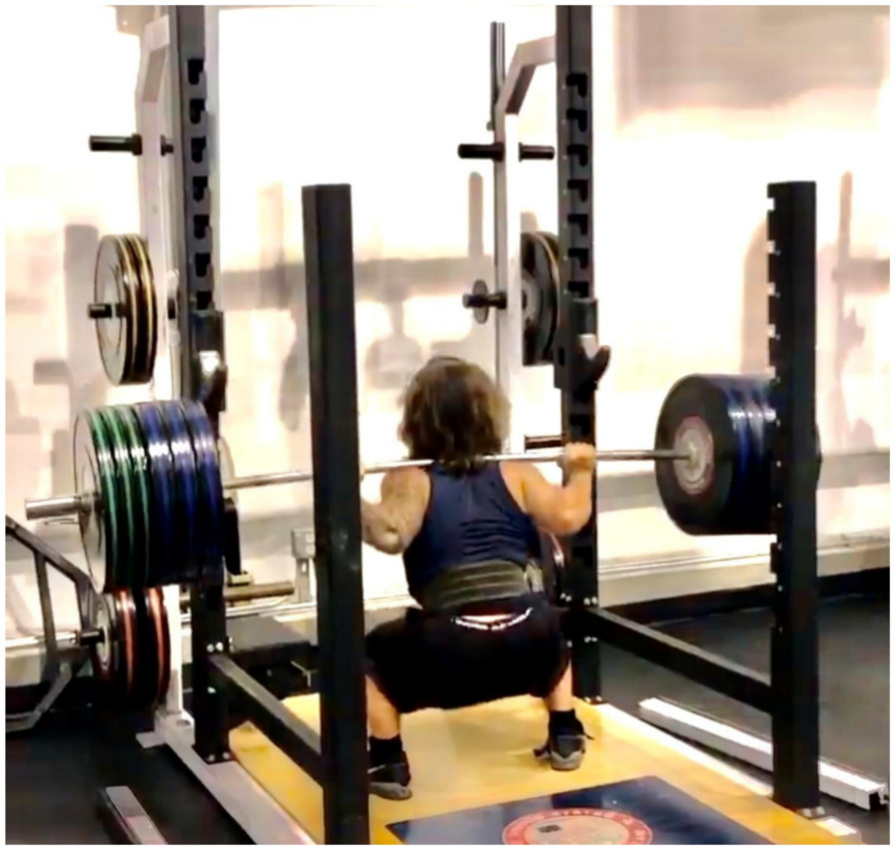
Squat-rack modification for an athlete with skeletal dysplasia (SD). The J-hooks and safety arms are lowered to 62 cm (barbell center height) to match Landry's shorter limb lengths, permitting an unrack/rerack path that remains within 5° of vertical and eliminates unnecessary shoulder elevation. Adjustable nylon spotting straps are set 2 cm below his maximal required squat depth, ensuring both safety and compliance with competition-specific depth criteria while avoiding excessive compressive loading during failed lifts.

**Figure 3:**
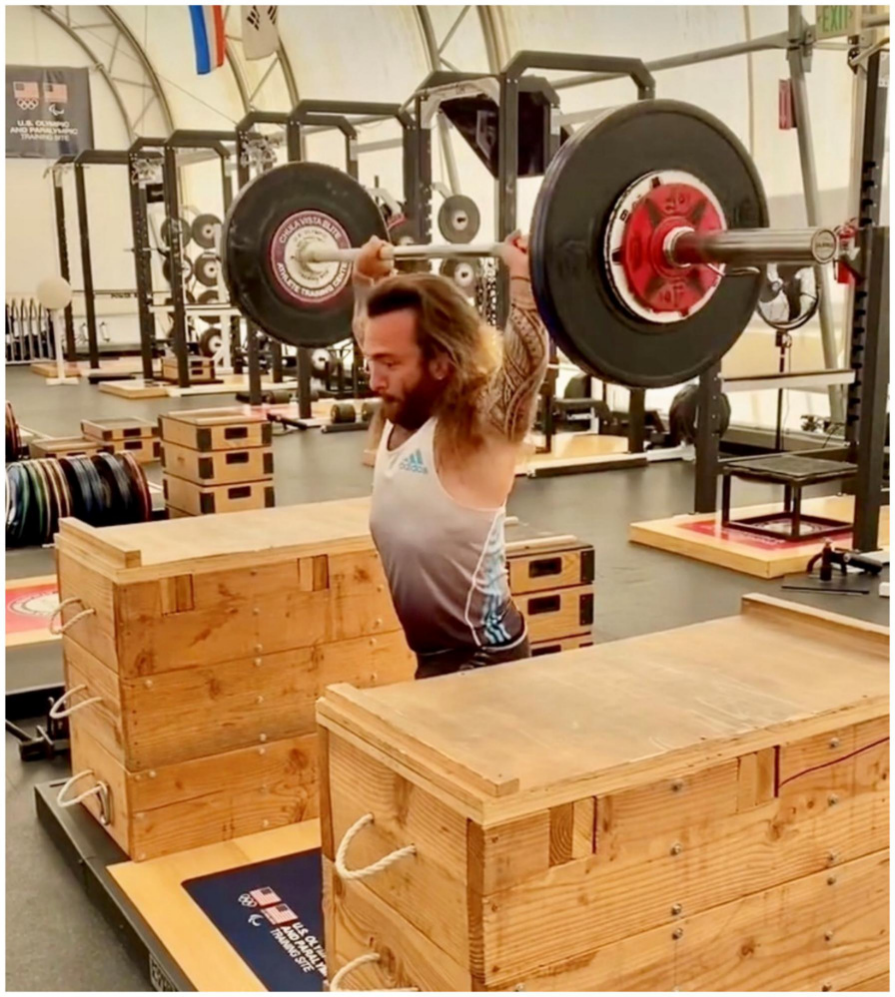
Box-elevated split jerk for overhead power development. Two 20 cm jerk boxes raise the barbell to mid-thoracic level in the start position. This height reduction shortens the concentric pull distance by ≈30%, allowing Landry to accelerate the load through the critical dip-and-drive phase without compromising triple-extension velocity. The setup also limits shoulder abduction beyond 135°, an important consideration for athletes with proportionally wider torsos and relatively shorter humerus, thereby reducing glenohumeral strain during repeated high-intensity sets. All adaptations were designed and implemented under the supervision of a Certified Strength and Conditioning Specialist in accordance with current Paralympic training guidelines (Vanlandewijck and Thompson 43).

For example, standard gym equipment such as benches may not accommodate the shorter limb length and stature of these athletes. In the case of the bench press, modifications such as placing boxes or blocks under the athlete's feet may be required to provide proper support and stability during the lift (Figure 1). These adaptations help ensure the athlete maintains correct body alignment and safety throughout the exercise. Similarly, squat adaptations (Figure 2) or the use of overhead jerk boxes (Figure 3) can be implemented to ensure exercises are performed safely and effectively, reducing the risk of injury while maximizing performance.

Coaches should be aware of the height and reach of equipment to avoid unnecessary strain or injury. Rather than viewing environmental constraints as limitations, they should be seen as opportunities to enhance the athlete's interaction with their surroundings. By utilizing specialized training aids and modifying drills, coaches can create an optimal training environment. This ensures physical safety while promoting an empowering training experience, building an athlete's confidence and encouraging athletic development (16). This adaptive approach can maximize output performance while also building a safe physical and mental environment for the athlete (24).

## 7 Task-specific training for athletes with SD in the shot-put

Designing effective training programs for Paralympic shot-put athletes with SD requires a biomechanically informed, scientifically grounded approach that addresses the distinct physical and physiological challenges posed by short stature. These athletes face distinct demands, including altered leverage, mobility limitations, and decreased muscle force generation. Focusing on optimizing core stability, upper body strength, and biomechanical adaptations is essential for enhancing performance in shot-put athletes with SD. A comprehensive review of adaptive strength and conditioning methods for athletes with physical impairments supports this approach, as personalized strategies are necessary for optimizing performance while minimizing injury risks (16).

## 8 Core stability

Core stability is foundational for shot-put athletes with SD due to their shorter limbs, which reduce leverage and impact the transfer of force through the kinetic chain (25). A strong and stable core is essential for stabilizing the torso during rotational movements, allowing for more efficient force transfer from the lower to the upper body, despite lower limb limitations (24). The rectus abdominis, obliques, and erector spinae muscles play critical roles in stabilizing the trunk and facilitating powerful rotational movements during the throw. Incorporating exercises such as medicine ball rotational throws, planks with rotation, and weighted sit-ups improves core strength, enhances kinetic chain efficiency, and ensures fluid force transmission throughout the shot-put motion (26). Additionally, strengthening these muscles helps reduce compensatory movements, which are common in athletes with biomechanical asymmetries.

## 9 Upper body strength

For athletes with SD, particularly those with limited lower body mobility, upper body strength becomes the primary determinant of shot-put performance (27). The pectoralis major, anterior deltoids, triceps brachii, and latissimus dorsi are key to generating the explosive force required for the arm strike during the final phase of the throw (28). This phase involves powerful extension and acceleration of the arm, making both concentric and eccentric muscle actions imperative for developing the necessary strength and speed. Progressive resistance training exercises, such as bench presses, overhead jerks, and triceps extensions, are critical for building the upper body power required to compensate for limited lower limb contribution (29). Strengthening scapular stabilizers, including the serratus anterior and rhomboids, is equally important for maintaining shoulder stability during forceful arm extension, a common concern for athletes with SD (30). Consequently, emphasis should be placed on controlled, upper body dominant exercises that enhance muscle power without compromising joint integrity. Medicine ball throws (overhead slams, rotational passes) and modified rebound push-ups have demonstrated particular effectiveness for developing the reactive strength necessary during the final acceleration phase of the throw.

## 10 Plyometric training

To develop explosive power, plyometric training (i.e., rapid eccentric-concentric muscle actions exploiting elastic energy and stretch reflexes) serves as a powerful tool for enhancing functional movement patterns and sport-specific skills in both rehabilitation and performance enhancement contexts (31, 32). In SD athletes with short limbs or reduced joint mobility, the upper body plyometric capacity, especially in the pectoralis major, triceps brachii, and anterior deltoids, becomes crucial for generating the reactive strength needed in shot-put final phase (33). While for lower limbs, a combination of high-intensity resistance training and Olympic lifting exercise variations aim to develop muscle power (34). The unique anthropometry of SD athletes requires modifications to traditional plyometric protocols. Shorter limb length decreases the moment arm available for force production (35), while potential joint hypermobility increases injury risk during high-impact landing phases (36). Landry displayed remarkable explosive strength, consistently generating countermovement-jump heights above 0.45 m, and his program strategically incorporated concentric-only jumps onto a 75 cm box to overload rate-of-force development while limiting eccentric strain, an evidence-based plyometric method aimed at transferring vertical impulse to shot-put release velocity (31).

## 11 Biomechanical adaptations

Given the unique anthropometry of athletes with SD, biomechanical adaptations are necessary to optimizing shot-put performance. The throwing motion requires precise coordination of the shoulder girdle, arm, and hand, with key muscle groups such as the pectoralis major, anterior deltoid, and triceps brachii driving the arm extension and release (37). The shorter limb lengths in athletes with SD require adjustments to optimize leverage, as these athletes have less mechanical advantage during the throw. To maximize shot velocity, the final stage of the throw should emphasize shoulder flexion, elbow extension, and an inside out wrist flick to impart proper spin and ensure the shot is released at the optimal angle for flight stability (11). Modifying release mechanics and implementing specialized training aids, such as weighted medicine balls, can help athletes develop more efficient throwing techniques (23, 38). Special exercises target the rapid elbow-extension, wrist-flick sequence, thereby retraining the neuromuscular firing pattern that governs release velocity and angle.

## 12 Task-specific shot-Put training

Task-specific training is crucial for developing the neuromuscular coordination and muscle memory required shot-put performance. Repetitive drills focusing on proper hip alignment, power position execution, and efficient release mechanics are vital for developing consistent and repeatable performance during competition. Developing a core strength is essential for stabilizing the torso during rotational movements while ensuring efficient force transfer between the lower and upper body (16). Core-focused exercises, such as medicine ball rotational throws, planks, and weighted sit-ups, enhance kinetic chain efficiency and minimize compensatory movement patterns (20).

Utilizing movement pattern specificity through drills allows athletes to focus on sequencing and timing their throw without the full load of a shot-put (11). These drills enhance proprioception and motor learning, allowing more efficient and explosive releases. Enhanced proprioception ensures athletes quickly adapt to competitive conditions, maintaining technical precision with pressure applied (21).

Hip pop drills are particularly effective for improving hip mobility and explosiveness, which generates power during the glide and rotational techniques in shot-put (25). While both techniques are used, most athletes, including Paralympian Hagan Landry, prefer the glide for its simplicity and efficiency in maximizing force transfer. Incorporating task-specific training enables athletes with SD to optimize shot-put technique and develop neuromuscular skills for peak performance. Medicine ball drills, focusing on release mechanics, can reduce overuse injuries by replicating shot-put mechanics while decreasing strain on the wrist, elbow, and shoulder while refining task-specific mobility. These drills maintain movement pattern specificity while lowering joint stress, ultimately facilitating technique refinement, improving motor control, coordination, and timing, and preventing injuries and muscular imbalances (11). Table 4 presents a series of task-specific shot-put drills designed to target fundamental elements such as power, balance, footwork, and release mechanics, aimed at enhancing overall performance.

**Table 4:** Task-specific shot-put drills.

Drill	Execution	Purpose
Hip Pop	The athlete initiates a controlled anterior pelvic tilt from the power position, driving hip extension while maintaining thoracic stability and scapular retraction. This coordinated motion maximizes force transfer through the kinetic chain while preserving upper body alignment.	Optimize hip extension through coordinated pelvic rotation while maintaining upper body stability. This enhances force generation and efficient energy transfer through the kinetic chain, improving shot-put performance.
Hip Pop Presses	Similar to the hip pop, the athlete initiates hip internal rotation, followed by shoulder flexion and elbow extension, completing the movement with a vertical press of the bar or dumbbell.	Enhances neuromuscular coordination between hip extension and upper limb force production, closely replicating the kinetic sequence of shot-put mechanics.
Overhead Bar Twists	The athlete maintains lower body stability through isometric contraction of the lower extremities while executing controlled trunk rotation in the transverse plane. The bar is held in overhead shoulder flexion, with scapular stabilization engaged to ensure proper alignment and balance throughout the movement.	Enhances rotational torque and core stability by engaging the obliques, transverse abdominis, and multifidus, which are critical for efficient force transmission through the kinetic chain during the throwing motion. This improved coordination maximizes power output and minimizes energy leakage, optimizing performance.
C-Position Reach Back	The athlete begins in the power position, with the hips in slight flexion and the knees bent, while maintaining a neutral spine. From this stance, the athlete initiates trunk rotation in the transverse plane, engaging the obliques and spinal rotators. Simultaneously, the athlete reaches posteriorly, extending the shoulder into the “C-position,” with scapular retraction and thoracic extension, contacting the wall to emphasize range of motion and stretch in the anterior kinetic chain.	Enhances glenohumeral joint flexibility and shoulder girdle mobility, promoting increased range of motion and scapulothoracic coordination. This is essential for optimizing the final phase of the shot-put release by maximizing the athlete's ability to achieve full extension and efficiently transfer kinetic energy into the projectile.
Seated Side Throws with Medicine Ball	The athlete sits on the floor with hips flexed and feet anchored, engaging the obliques, transverse abdominis, and spinal rotators to generate rotational force in the transverse plane. The athlete then explosively throws a medicine ball laterally against the wall, emphasizing eccentric and concentric core activation to enhance rotational power and neuromuscular coordination for force transfer.	Improves core stability by activating the transverse abdominis and multifidus, increases rotational torque through oblique and spinal rotator engagement, optimizes kinetic chain efficiency for enhanced force transfer, and enhances neuromuscular coordination between the core, hips, and shoulders.
Medicine Ball Drops	In this plyometric action, the athlete lies in a supine position, and the partner performs a medicine ball drop to enhance upper body acceleration by eccentrically absorbing force and transitioning into concentric shoulder and arm extension for explosive power development.	Enhances neuromuscular coordination between the upper limbs and core, improves stretch-shortening cycle efficiency for rapid force production, and develops reflexive muscular activation to increase upper body speed and explosive power in shoulder flexion, elbow extension, and core stabilization.

## 13 Dynamic daily undulating periodization for paralympic athletes

Daily undulating periodization (DUP) is an evidence-based model of periodization that alternates training variables such as volume and intensity on a frequent basis, often daily, to promote adaptation and performance (39). Unlike traditional linear periodization, these variables are adjusted gradually over extended periods, DUP allows for greater flexibility and responsiveness to an athlete's needs during training (40). Research highlights the efficacy of DUP in enhancing strength and performance by incorporating frequent fluctuations in load and intensity, making it effective for athletes with complex training demands, such as Paralympic athletes (26). The inclusion of DUP ensures that an athlete's training plan is dynamic, preventing plateaus while allowing optimal adaptation based upon individual constraints.

## 14 The need for dynamic periodization in training paralympic athletes

For Paralympic athletes, a dynamic approach to periodization is crucial due to fluctuating physiological and psychological demands imposed by disabilities and competitive environments (16). Traditional periodization models often rely on long-term, fixed progressions of intensity and volume, which fail to accommodate the fluid constraints of Paralympic athletes (26). These constraints may include mobility variation, changes in physical capacity due to injury, or use of specific adaptations like assistive devices (22). Thus, adopting a more flexible and adaptive approach, such as DUP, is imperative for optimizing performance while mitigating injury risk (26).

The Periodization of Skill Training (PoST) framework further advances this approach by integrating skill acquisition with physical conditioning (21, 41). This framework emphasizes the development of technical proficiency alongside physical attributes, facilitating a comprehensive periodization strategy that addresses both motor learning and physical development (41). This dual focus ensures that athletes are not only physically prepared but technically proficient, which is crucial during competitive seasons which demands physical and skill peak simultaneously (42). Because this investigation centered on a single athlete with SD, its generalizability across impairment classes and populations is limited. Moreover, while the OHB shot-put test provided useful insight into neuromuscular trends (see Table 2), the absence of a concurrent control group and additional biomarkers of fatigue (e.g., hormonal, inflammatory) constrain our ability to definitively distinguish adaptation from overtraining.

## 15 Conclusion

This study supports the premise that a *constraints-based* strength and conditioning framework, when combined with dynamic periodization and a multidisciplinary support team, can meaningfully elevate performance in Paralympic athletes with short stature (SD). By systematically mapping individual, task, and environmental constraints, the coaching staff can tailor training prescriptions with precision, rather than relying on generic templates. Embedded within this framework, the Overhead Back (OHB) shot-put test was used as both a fitness checkpoint and early warning system: over the 24-week preparatory mesocycle, throw distances rose from 12.00 m to 14.10 m (see Table 2), illustrating not only performance progression but also the utility of the OHB measure for monitoring fatigue, plateauing, or overreaching. This dual role of the OHB testing metric, enhancing adaptation and safeguarding against maladaptive loading, underscores the value of integrating objective performance diagnostics into routine programming, especially for populations with altered biomechanics and injury sensitivity. More broadly, our findings suggest that performance optimization in adaptive sport demands a synthesis of individualized constraint analysis, responsive periodization, and continuous feedback loops across physical and psychological domains. Future research should extend this paradigm to other impairment classes and sport contexts, employing multicenter collaborations to test scalability. Critical next steps include validating the sensitivity and specificity of in-field diagnostics like the OHB test, refining algorithms for recovery/load modulation, and quantifying long-term injury rates relative to constraint-based programming. By doing so, the field moves closer to a robust, evidence-informed model of elite Paralympic preparation.

## Data Availability

The original contributions presented in the study are included in the article/supplementary material, further inquiries can be directed to the corresponding author/s.
